# Preoperative neurocognitive function as an independent survival prognostic marker in primary glioblastoma

**DOI:** 10.1093/nop/npad027

**Published:** 2023-05-11

**Authors:** Evangelia Liouta, Christos Koutsarnakis, Spyridon Komaitis, Aristotelis V Kalyvas, Evangelos Drosos, Juan M García-Gómez, Javier Juan-Albarracín, Vasileios Katsaros, Lampis Stavrinou, George Stranjalis

**Affiliations:** 1st Department of Neurosurgery, National and Kapodistrian University of Athens, Evangelismos Hospital, Athens, Greece; Hellenic Center for Neurosurgical Research “Prof. Petros Kokkalis,” Athens, Greece; 1st Department of Neurosurgery, National and Kapodistrian University of Athens, Evangelismos Hospital, Athens, Greece; Athens Microneurosurgery Laboratory, Grupo de Informática Biomédica (IBIME), Instituto de Aplicaciones de las Tecnologías de la Información y de las Comunicaciones Avanzadas (ITACA), Universitat Politècnica de València, Valencia, Spain; 1st Department of Neurosurgery, National and Kapodistrian University of Athens, Evangelismos Hospital, Athens, Greece; Athens Microneurosurgery Laboratory, Grupo de Informática Biomédica (IBIME), Instituto de Aplicaciones de las Tecnologías de la Información y de las Comunicaciones Avanzadas (ITACA), Universitat Politècnica de València, Valencia, Spain; 1st Department of Neurosurgery, National and Kapodistrian University of Athens, Evangelismos Hospital, Athens, Greece; Athens Microneurosurgery Laboratory, Grupo de Informática Biomédica (IBIME), Instituto de Aplicaciones de las Tecnologías de la Información y de las Comunicaciones Avanzadas (ITACA), Universitat Politècnica de València, Valencia, Spain; 1st Department of Neurosurgery, National and Kapodistrian University of Athens, Evangelismos Hospital, Athens, Greece; Athens Microneurosurgery Laboratory, Grupo de Informática Biomédica (IBIME), Instituto de Aplicaciones de las Tecnologías de la Información y de las Comunicaciones Avanzadas (ITACA), Universitat Politècnica de València, Valencia, Spain; Athens Microneurosurgery Laboratory, Grupo de Informática Biomédica (IBIME), Instituto de Aplicaciones de las Tecnologías de la Información y de las Comunicaciones Avanzadas (ITACA), Universitat Politècnica de València, Valencia, Spain; Athens Microneurosurgery Laboratory, Grupo de Informática Biomédica (IBIME), Instituto de Aplicaciones de las Tecnologías de la Información y de las Comunicaciones Avanzadas (ITACA), Universitat Politècnica de València, Valencia, Spain; Department of Radiology, General Anti-Cancer and Oncological Hospital of Athens “St. Savvas”, Athens, Greece; 2nd Department of Neurosurgery, National and Kapodistrian University of Athens, ATTIKO Hospital, Athens, Greece; 1st Department of Neurosurgery, National and Kapodistrian University of Athens, Evangelismos Hospital, Athens, Greece; Hellenic Center for Neurosurgical Research “Prof. Petros Kokkalis,” Athens, Greece; Athens Microneurosurgery Laboratory, Grupo de Informática Biomédica (IBIME), Instituto de Aplicaciones de las Tecnologías de la Información y de las Comunicaciones Avanzadas (ITACA), Universitat Politècnica de València, Valencia, Spain

**Keywords:** cognition, executive functions, GBM, overall survival, prognosis

## Abstract

**Background:**

Aim of the present study is to investigate whether preoperative neurocognitive status is prognostically associated with overall survival (OS) in newly diagnosed glioblastoma (GBM) patients.

**Methods:**

Ninety patients with dominant-hemisphere IDH-wild-type GBM were assessed by Mini Mental Status Exam (MMSE), Trail Making Test (TMT) A and B parts, and Control Word Association Test (COWAT) phonemic and semantic subtests. Demographics, Karnofsky Performance Scale, tumor parameters, type of surgery, and adjuvant therapy data were available for patients.

**Results:**

According to Cox proportional hazards model the neurocognitive variables of TMT B (*P* < .01), COWAT semantic subset (*P* < .05), and the MMSE (*P* < .01) were found significantly associated with survival prediction. From all other factors, only tumor volume and operation type (debulking vs biopsy) showed a statistical association (*P* < .05) with survival prediction. Kaplan Meier Long rank test showed statistical significance (*P* < .01) between unimpaired and impaired groups for TMT B, with median survival for the unimpaired group 26 months and 10 months for the impaired group, for COWAT semantic (*P* < .01) with median survival 23 months and 12 months, respectively and for MMSE (*P* < .01) with medial survival 19 and 12 months respectively.

**Conclusions:**

Our study demonstrates that neurocognitive status at baseline—prior to treatment—is an independent prognostic factor for OS in wild-type GBM patients, adding another prognostic tool to assist physicians in selecting the best treatment plan.

Glioblastoma (GBM) is the most common malignant primary brain tumor in adults characterized by significant neurological morbidity and poor prognosis. Although considerable progress has been made over the past years in the treatment of glioblastoma, prognosis remains poor, with the majority of clinical trials reporting a median overall survival (OS) of less than 2 years.^1^ Nonetheless, there is a notable variability in OS among patients, ranging from few months to more than 5 years.^2^ This may be attributed to parameters falling into 1 of the 3 main categories: The first one includes tumor-based characteristics such as location,^3^ volume,^4^ as well as genetic characteristics^5,6^; the second one includes treatment-based parameters such as surgery versus biopsy, extension of resection and adjuvant therapy while the third one is associated with patient-based characteristics such as age and clinical performance status.^7^

Patient performance status is one of the most common parameters employed as a prognostic factor by the majority of clinical studies, but also as a criterion upon which treatment management is based. The Karnofsky Performance Status (KPS) scale is the most widely used in neurooncological studies and categorizes patients according to their level of independence. However, recent studies highlight the fact that KPS does not accurately represent the patient’s neurological and neurocognitive status, and this becomes increasingly important considering that neurocognition is the most negatively affected clinical parameter in GBM cohorts, with the majority of the patients presenting neurocognitive impairments at the time of diagnosis.^8^

In this content, a number of studies have recently sought to investigate the prognostic role that neurocognitive status may play in the overall survival of newly diagnosed GBM patients by employing measures reflecting brain functioning ie, cognitive assessment. The first evidence for the prognostic role of neurocognition in OS of patients with recurrent malignant gliomas is provided by Mayers et al. (2000).^9^ Klein et al. (2003) were the first to report the prognostic value of neurocognitive impairment in survival time in a sub-group of older patients with newly diagnosed glioblastoma.^10^ It should be noted; however, that no specific domain of cognitive dysfunction (perception, memory, attention, and executive ability) was linked to survival time, but rather the presence of dysfunction itself. In 2008, Gorlia and colleagues reinforced the prognostic importance of neurocognitive status by supporting that global cognition—as measured by MMSE—was independently associated with OS in patients participating in the EORTC and NCIC trial 26981-22981/CE.3.^11^ More recently, in an effort to further elucidate the prognostic role of domain-specific cognitive dysfunction in survival of patients diagnosed with newly diagnosed GBM, Johnson et al. (2012) were the first to demonstrate a significant association between performance on attention and executive functions and survival time.^12^ Later on, executive functions’ performance status was reconfirmed as an independent prognostic factor for OS in GBM, with research to suggest that the combination of executive dysfunction and mood disorders (depressive symptoms) is linked to even shorter OS in GBM patients, more so than the executive functions’ impairment alone.^13^

In all the aforementioned studies, neurocognitive assessment is characterized as measurement at “baseline.” In the content of neurooncological trials; however, baseline assessment refers to pre-radiation and chemotherapy time points, yet at a post-operative time. This methodological characteristic may result in underestimation of the prognostic role of neurocognitive status in GBM survival for the following reasons: Firstly, it is well-known that surgery can affect cognition and may result in new cognitive impairment, deterioration of a preexisting one or even in amelioration in cases where regional dysfunction is a result of reversible causes such as edema or compression of brain structures.^8^ Furthermore, different operation techniques (ie, biopsy, total-subtotal excision) in different brain regions may differently affect patients’ cognition. Secondly, apart from the post-operative cognitive sequelae per se, patients often exhibit increased fatigue for a few days/weeks after surgery, which in turn may negatively affect performance on cognitive testing. Thirdly, the time of “baseline” assessment in the past studies usually precedes the time (around 3 months), where neurocognitive status can be considered stable. Forth, patients included in chemo-radiation trials may have been subject to a referral effect according to which there might have been a preselection of patients who underwent cognitive assessment based on their level of disability.^14^ It should be also noted that none of the aforementioned studies has taken into consideration the impact of the tumor per se on cognitive function, which is considered the greatest one compared to other factors. Knowing the extent of neurocognitive impairment prior to any intervention and examining whether the preoperative neurocognitive status can have an independent prognostic value in the OS of patients harboring a GBM could offer clinicians another tool to aid decision-making. Only limited number of studies have recently supported the independent relationship between preoperative cognition and survival in diffuse glioma (II to IV) patients undergoing awake craniotomy.^15,16^ The aim of the present study is to investigate whether baseline preoperative neurocognitive function status is prognostically associated with OS in newly diagnosed GBM patients.

## Methods

### Participants

**Figure 1.**
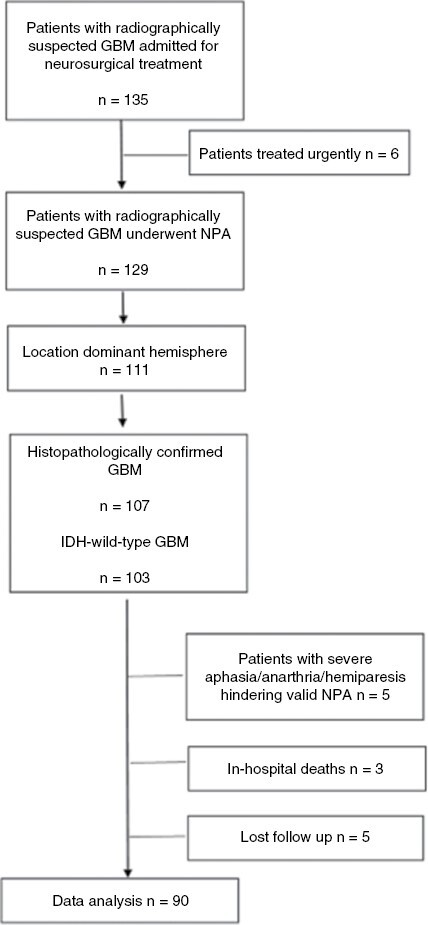
Patient selection flow chart.

123 patients with radiographically suspected GBM were admitted for neurosurgical treatment to the University Neurosurgical Clinic of “Evangelismos” Hospital in Athens, Greece, between 2016 and 2019 and were referred for neuropsychological assessment prior to operation. As the majority of tumors were located in the dominant hemisphere, we excluded non-dominant hemisphere cases in order to homogenize our sample. Therefore, our population consisted of a consecutive series of patients with left/ dominant hemisphere suspected GBM. After histopathological confirmation of primary IDH-wild-type GBM, exclusion of patients with insufficient cooperation, in-hospital deaths, and those lost at follow-up, a total of 90 patients were included in the present study (see Figure 1 for patient selection flow chart). Demographic data (age, gender), clinical characteristics (KPS), tumor parameters (location, volume), number and type of surgeries (debulking vs. biopsy), adjuvant therapy (radiotherapy, chemotherapy), and OS data were available for all enrolled participants. OS was obtained from the Neurosurgical Department’s electronic database. The time between admission at the hospital for operative treatment and the death recorded in our department’s database was defined as OS. Censoring was defined as the period between admission at the hospital for surgery and the last follow-up. Lost follow-up cases are considered the ones that discontinued communication after operation. Consent forms were obtained from all participants prior to testing. The study was approved by the Research Ethics Committee of “Evangelismos” General Hospital. This study was performed in line with the principles of the Declaration of Helsinki.

### Neurocognitive Assessment

Neurocognition was prospectively assessed with the following battery of standardized neuropsychological tests: Mini Mental Status Exam (MMSE) for global cognitive functioning,^17^ Trail Making Test (TMT) -part A to test speed processing and part B for complex attention executive functioning^18^ and verbal fluency test (COWAT) phonemic and semantic subtests for language-executive functioning.^19^ Corrected for age and education scores were calculated and were used in our analyses for TMT and COWAT tests and scores ≥- 1.5SD from the healthy norms were considered clinically pathological. MMSE performance was considered normal when the score was ≥26/30.^20^ The majority of our patients underwent a bedside assessment. Paper-and-pencil tests were employed with their duration not to exceed 20–25 minutes. Dominant hemisphere was determined by the combination of the following data: (1) Handedness Edinburgh Inventory, (2) clinical symptoms, and (3) fMRI for language laterality and localization.

### Tumor Characteristics

Preoperative brain Magnetic Resonance Imaging (MRI) scans were obtained 7.3 ± 2.5 days before preoperative neurocognitive assessment. MRI scans were reviewed and tumor location was categorized according to the cerebral lobe the lesion occupied (frontal, parietal, temporal [including insula], and occipital). Tumors that occupied more than one lobe were categorized according to the tumor’s largest volume location. MRI volume calculations were performed using the ONCO habitats software system (https://www.oncohabitats.upv.es).^21^ For more information see https://www.oncohabitats.upv.es.^16^ Lesion volumetry was conducted in a blind fashion to clinical stratification. A histopathological diagnosis of IDH-wild-type GBM was determined according to WHO 2016 criteria.

### Statistical Analysis

Data were analyzed using the SPSS software package (IBM SPSS Statistics for Windows, Version 26.0, Armonk, NY: IBM Corp). Descriptive statistics for demographic, tumor, clinical, and neuropsychological parameters were presented as means and SDs for continuous variables or frequencies when dichotomous variables were employed. Multivariate and Chi-Square analyses with factors the impaired versus non-impaired groups in MMSE, TMT A, TMTB, COWAT phonemic, and semantic subsets and dependent variables age, gender, KPS, tumor volume, tumor location, and type of surgery and were also performed. Survival analysis was conducted using Cox proportional hazards model. Our Cox model included all neurocognitive measures (MMSE, TMT A, TMT B, and COWAT phonemic and COWAT semantic) as well as all relevant demographical (age and gender) and clinical (KPS, tumor volume, tumor location, and type of operation (debulking vs. biopsy) factors. We did not include second operation in our model as patients that underwent reoperation were selection biased based on prior history, making thus this parameter a poor candidate as an independent factor in survival analysis. Finally, adjuvant therapy was not included in our model as (1) only a minority (7 out of 90) of patients did not receive it or discontinued it due to early severe side effects, making it thus a very poor candidate as independent factor in survival analysis due to powerless statistics, and (2) all patients received adjuvant therapy underwent the same protocol (Stupp therapy). Results from neuropsychological tests were analyzed as dichotomized -by deficit- variables. Subsequently, a Kaplan–Meier survival analysis was conducted between cognitively impaired and unimpaired patients for each of the neuropsychological tests found significant in the Cox proportional hazards model analysis.

## Results

### Demographic and Clinical Parameters

Ninety patients with newly diagnosed dominant hemisphere primary IDH-wild-type GBM were neuropsychologically assessed preoperatively. At the time of analysis, 15 of the 90 patients (16.7%) were confirmed to be censored (alive). Descriptive results for demographic and clinical characteristics are presented as means or frequencies in Table 1.

**Table 1.** Demographic and Clinical Data

Characteristics	Mean ± (Range)	*N* (%)
Age	58.8 ± 8.9 (48-79)	
Male sex		54(60)
KPS≥90		77(85.6)
Left DH		86(95.6)
Tumor location		
Frontal		35 (38.9)
Temporal (incl. insula)		42(46.7)
Parietal		11 (12.2)
Other		2 (2.2)
Tumor volume		38.0 ± 32.4(2.20-128.0)
Operation type		
Debulking		72(80)
Biopsy		18(20)
Adjuvant therapy(Stupp)		83(92.3)

KPS, Karrnofsky Performance Scale; DH, Dominant Hemisphere; ±, Standard Deviation; Age mean values represent years; Tumor volume values represent cm^3^.

### Neurocognitive Results

Descriptive statistics for neuropsychological measures are presented as means and frequencies of impairment in Table 2. More than half of the patients showed impairment in global cognition and executive function, half of the patients exhibited deficits in verbal fluency and one in three patients performed pathologically in speed processing.

**Table 2.** Performance on Neurocognitive Measures

Domain	Test	Mean ±	Impaired %
Speed processing	TMT A	−1.23 ± 0.97	27.9
Executive function/attention	TMT B	−1.59 ± 1.22	62.1
Language	COWAT phonemic	−1.63 ± 1.25	50.7
	COWAT semantic	−1.83 ± 1.35	45.8
Global cognition	MMSE	22.4 ± 5.29	68.6

TMT, Trail Making Test; COWAT, Controlled Word Association Test; MMSE, Mini Mental Status Exam; result values for all test but MMSE represent standard deviation from the norms; ±, standard deviation

### Neurocognitive and Demographic/Clinical Parameters Relationship

Multivariate analyses for continuous variables showed that impaired groups in MMSE, TMT A, TMT B, and COWAT phonemic and semantic subsets had a larger tumor volume (*P* < .05) than their respectively non-impaired ones. Age on the other hand did not show significant difference (*P* > .05) between the impaired-non-impaired groups in any of the cognitive measures. Fisher exact test for categorical variables showed that KPS was significantly different (*P* < .05) between the TMT A groups with the impaired one to have a lower KPS than the normal one. All other comparisons between cognitively impaired versus non-impaired groups and demographic/clinical parameters did not reveal any statistical significance (*P* > .05). Please see Table 3.

**Table 3.** Comparisons (*P*-values) of Demographic/Clinical Parameters Between Impaired and Non-impaired Groups for Each Neurocognitive Variable

Impaired vs. Non-impaired Groups					
	MMSE	TMT A	TMT B	COWAT Phonemic	COWAT Semantic
Age	0.100	0.858	0.127	0.362	0.067
Gender	0.237	0.109	0.655	0.062	0.387
KPS	0.057	0.046*	0.119	0.065	0.234
Tumor location	0.429	0.635	0.253	0.086	0.071
Tumor volume	0.005**	0.029*	0.026*	0.036*	0.001**
Surgery type (debulking vs. surgery)	0.059	0.481	0.417	0.387	0.113

*, statistical significance at 0.05 level; **, statistical significance at 0.01 level; *P*-values for continuous variables (age, tumor volume) computed by multivariate analysis and for dichotomous variables by x^2^ analysis.

### Survival Analyses

According to Cox proportional hazards model the neurocognitive variables of TMT B (*P* < .01), COWAT semantic subset (*P* < .05), and the MMSE (*P* < .01) were found significantly associated with survival prediction. TMT A and COWAT phonemic subset did not show any association with survival prediction (*P* > .05). From all other factors only tumor volume and operation type (debulking vs. biopsy) showed a statistical association (*P* < .05) with survival prediction. Age, gender, KPS, and tumor location were not found significant predictors of survival. Results can be seen in Table 4.

**Table 4.** Cox Proportional Hazards Model for Cognitive, Demographic and Clinical Factors

Factors	Coefficient (B)	SE	HR (95% CI)	*P*-value
Cognitive				
TMT A	−1.978	1.159	0.014–1.341	.088
TMT B	−4.904	1.357	0.001–0.106	<.01**
COWAT phonemic	−0.705	1.142	0.053–4.631	.537
COWAT semantic	−3.105	1.551	0.002–0.937	<.05*
MMSE	−0.805	0.289	0.253–0.788	<.01**
Demographic				
Age	0.051	0.043	0.968–1.145	.231
Gender	0.129	0.725	0.275–4.717	.859
Clinical				
KPS	−1.557	1.043	0.027–1.630	.136
Tumor Volume	−0.025	0.012	0.953–0.998	.037*
Tumor lobe	−0.57	1.032	0.125–7.146	.956
Operation Type	−2.432	1.223	0.351–1.493	.045*

*Significance level at *P* = .05; ** Significance level at *P* = .01.

**Figure 2.**
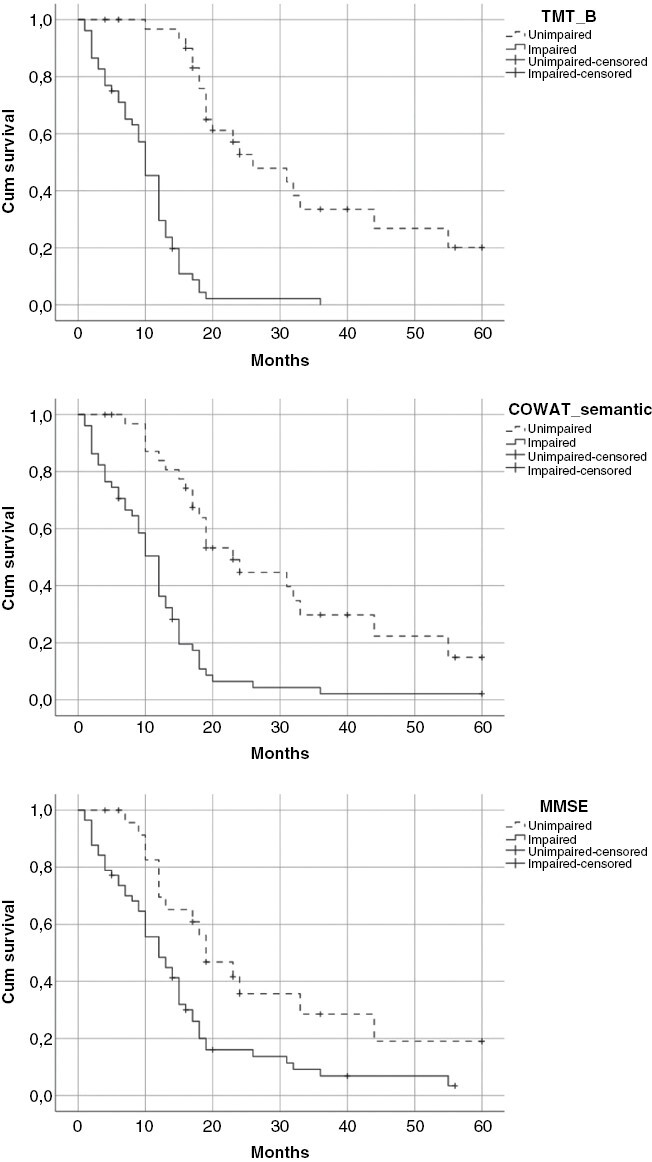
Kaplan–Meier survival plots by impairment on neurocognitive measures of Trail Making Test B subset (*P* < .01), COWAT semantic subset (*P* < .01) and Mini Mental Status Exam (*P* < .01).

Kaplan–Meier survival curves by impairment for TMT-B, COWAT semantic, and MSSE test are presented in Figure 2. Long rank test showed statistical significance (*P* < .01) between TMT-B unimpaired and impaired groups for OS: Median survival for the unimpaired group was 26 months and 10 months for the impaired group. For COWAT semantic test median survival was 23 months for the unimpaired group and 12 months for the impaired group. Finally, for MMSE medial survival was 19 and 12 months for unimpaired and impaired groups, respectively.

## Discussion

Aim of our study was to investigate the prognostic value of preoperative neurocognitive function status in OS of newly diagnosed primary IDH-wild-type GBM patients. To homogenize our sample, our study included only patients with dominant hemisphere tumors. Cognitive status was found to be an independent prognostic factor of OS in patients with dominant hemisphere primary GBM. In general, our study confirms outcomes of the existing literature supporting the prognostic role of clinical neurocognitive status in OS of patients harboring a GBM^10–13,22,23^ while it extends previous scarce research^15,16^ by providing evidence for the prognostic value of neurocognitive status in OS at an early point, prior to any treatment.

As emphasized by neuropsychological studies, KPS—usually used as a measurement of oncological patients’ clinical status—has not been designed for brain tumor patients and subsequently does not accurately reflect brain (dys)function. In this context, MMSE, assessing global cognition at a pre-radiotherapy/chemotherapy point, has been employed by a number of large sample neurooncological studies over the last years as a potential clinical prognostic factor in GBM survival. MMSE has been included as independent prognostic factor in nomograms for predicting survival of GBM, with a score of ≥27 to correlate with improved survival outcome.^11^ Similarly, patients with higher MMSE scores had longer progression-free and OS than those with lower scores in a more recent trial that investigated the effect of short-course radiation plus temozolomide in survival of elderly patients with GBM.^14^ On the other hand, Tanzilli et al. (2020) did not find any relationship between MMSE and OS scores, obtained three weeks postoperatively and prior to radiotherapy and/or chemotherapy, in elderly patients with GBM.^22^ It should be noted however that in this study the majority of the patients (80%) did not have deficits on global functioning (MMSE)—a rather uncommon finding in GBM patients—and that the authors did not mention the MMSE score range under which patients had been categorized as having a deficit or not. In the present study, we found that global cognitive status (MMSE) was independently associated with OS; patients with normal MMSE score reached a 19-month medial OS while patients with abnormal score reached a 1-year medial OS. Our results thus are in line with the ones found in the 2 large-scale studies and extend the literature by supporting the notion that tumor-induced deficits on global cognition—at a time point before any treatment—are independently associated with OS in primary GBM patients.

Apart from global cognition, executive functions measured by TMT-B and verbal fluency assessed by COWAT were also found to be independent predictors of OS in GBM patients. In fact, TMT-B and semantic COWAT unimpaired patients had a more than double survival time compared to impaired ones, thus outweighing the survival time difference predicted by MMSE. Executive functions, although considered frontal ones, rely on a complicated—gray and white matter—network and their integrity is greatly affected in patients diagnosed with tumors. Thus, executive function tasks such as TMT-B and COWAT have been selected among other tests by the International Cognition and Cancer Task Force as key measures for clinical monitoring in brain tumor and cognition studies.^23^ Preliminary evidence that the domain of executive function is associated with survival in newly diagnosed patients with glioblastoma was offered a decade ago. Johnson et al.^12^ were the first to demonstrate that TMT-B and COWAT scores obtained prior to adjuvant therapy were independently associated with OS in 91 patients with newly diagnosed GBM. Later on, these results were reconfirmed by the same research team in a study that addressed the relation of survival and executive function (measured by TMT-B) in combination with depressive symptoms.^13^ More recently, patients’ performance on the Stroop test, another measure of executive function -attention, three weeks postoperatively, was found to be an independent prognostic factor for OS in GBM.^24^ Lastly, only 2 studies have addressed the predictive value of executive functions prior to surgery in GBM survival; The first one^15^ supported that impairments in executive functioning and memory domains are significantly and independently correlated with survival in diffuse (II-IV) gliomas. The second one examined the progression-free survival (PFS) at 6 months and not the overall survival in GBM.^25^ According to Lee et al., TMT-B was the only, among other clinical and cognitive factors, independent predictor for PFS-6, with the median PFS for TMT-B poor performers to be 17 weeks in contrast to median PFS for TMT-B good performers that had not been reached at 6 months. Conclusively, the present study reinforces the prognostic value of executive functions testing reported by previous research.

Although an association between mortality and neurocognitive functioning has been shown in patients with brain malignancy, the causal relationship between cognitive impairment and survival of patients diagnosed with GBM remains poorly understood. Previous authors^26^ have hypothesized that tumor-related cognitive deficits are significantly associated with pathological factors such as IDH mutation in that faster-growing tumors may allow less time for plasticity in contrast to low-grade gliomas. What´s more, IDH-mutant malignant astrocytomas have been shown to be more amenable to surgical resection than IDH-wild-type tumors, highlighting a possibly more infiltrative pattern of the latter.^27^ A second hypothesis is that of treatment bias, with physicians pursuing more aggressive treatment in patients with good neurocognitive function, thus leading to a better prognosis.^25^ As our neuropsychological evaluation took place prior to operation without affecting physicians’ therapeutic plan, our results are in support of the first hypothesis.

It is noteworthy that from all other well-known factors, only tumor volume and type of surgery (resection vs. biopsy) were found to predict OS in our study. Surprisingly, although tumor size is considered to have a prognostic value, the majority of previous studies investigating cognition as a predictor of survival in GBM did not account for tumor size in their prediction models,^11,12,14^ while others that did, supported that tumor volume is not a predictor for survival; it should be noted however that these studies were referred to a mixed sample of grade III and IV gliomas^9^ and to PFS-6, respectively.^25^ Hansen et al.s (2018) on the other hand reported that tumor diameter was an independent predictor for OS as well as cognitive impairment, but no specific cognitive function was mentioned.^28^ Only a recent study^15^ provided evidence for executive functions as an OS prognostic marker independently from tumor volume. Therefore, our study extends previous limited research by showing that both global cognition and executive functions are—independent from tumor volume—prognostic factors for OS in newly diagnosed GBM patients.

As per the type of surgery, we observed a statistical significance in OS when we compared resection versus biopsy as expected. Previous similar to our studies have also included the type of surgery as an independent factor in multivariate predictive models^9,12,13,22^ reporting no association with OS. However, in these studies, biopsy versus subtotal versus total resection approach was adopted rather than biopsy versus debulking. The fact that a minimum of 78% reduction in tumor volume is necessary to increase survival and that the cumulative benefit increases with more complete resections of up to 98%^29,30^ make us suspect that methodological issues in previous studies ie, gross (macroscopical) categorization of total and subtotal resections, may account for not observing a predictive role for the type of surgery.

Finally, it should be mentioned that established prognostic factors such as the patient’s age and KPS were not found to be significant predictors of OS in our sample. With reference to age, although some studies are in support of its prognostic value,^10,12,28^ others have found that when cognitive status is included in prognostic models, age is not a predictor of OS in GBM.^13,22^ In addition, studies employing age-stratified samples report that it is the clinical status and the comorbidities that increase with age which consist of significant prognostic elements in OS and not the age per se.^31,32^ Conclusively, the fact that the majority of our patients were in good clinical condition—due to assessment at a rather early point—may explain the nonsignificant effects of age in OS of our sample. In regards to KPS, despite the fact that it is considered a classic survival prognostic factor,^22,24^ few studies^12,32^ are in line with our results and have not found KPS as significant predictor of OS in wild-type GBM. Nonetheless, it could be that our outcome is explained by methodological issues, such as the fact that the majority (85%) of our sample had KPS >90, as opposed to other studies that did find KPS to be an independent prognostic factor for OS.

In overall, our study demonstrates that neurocognitive status at baseline—prior to treatment—is an independent prognostic factor for OS in primary IDH-wild-type GBM patients. Our outcomes stress the need for the incorporation of neuropsychological assessment in clinical exams of patients with GBM when they are admitted to the hospital for further treatment. Knowing the neurocognitive status of a patient preoperatively would add, along with other factors, a prognostic tool that in turn could assist physicians in selecting the best treatment plan.

Advantages of the present study include (1) the baseline time point of assessment, avoiding thus any treatment-induced confounding variables as well as a possible selection bias that may be observed in homologous studies employing patients referred for radio/chemotherapy, (2) a relative homogeneity of tumors in terms of location and histopathology, and (3) the fact that patients were assessed by the same neuropsychologist and operated by the same neurosurgeon avoiding thus interobserver validity issues.

At the same time, certain limitations characterize our study: as the majority of patients referred for neuropsychological assessment had a dominant hemisphere tumor, we preferred to exclude patients with lesion in the non-dominant hemisphere; thus, our results refer mainly to patients with a left primary GBM and one should be cautious to generalize the results of this study to patients harboring a right-hemisphere primary GBM. Another limitation of our study is that we excluded patients with insufficient cognitive-motor cooperation and patients who did not receive neuropsychological assessment due to urgent treatment. Thus, our results may not apply to all patients with dominant hemisphere GBM but only to those undergoing semi-elective surgery. Furthermore, although we homogenized our sample in terms of IDH mutation, other genetic factors were not taken into account due to their rarity such as non-canonical mutations (although the latter has not been associated with a different survival profile).^33^ Finally, as our cohort includes patients from 2016 to 2019, we have not been able to analyze molecular parameters incorporated into the WHO 2021 taxonomy.^34^ Future research should investigate whether new—according to the 2021 WHO CNS tumor classification—gene and molecular alterations (TERT promoter, chromosomes 7/10, and EGFR for GBM IDH- wild type) influence cognitive performance through mechanisms that include perturbation of neuronal communication. Lastly, in the present study, we did not control for O^6^-methylguanine-methyltransferase (MGMT) promoter methylation status. As MGMT promoter methylation status is not only prognostic in wild-type GBMs,^35,36^ but also predictive of glioblastoma response to treatment with temozolomide,^37^ our lack of controlling it as confounding factor may have affected our results.
